# ChIP-seq analysis of genomic binding regions of five major transcription factors highlights a central role for ZIC2 in the mouse epiblast stem cell gene regulatory network

**DOI:** 10.1242/dev.143479

**Published:** 2017-06-01

**Authors:** Kazunari Matsuda, Tomoyuki Mikami, Shinya Oki, Hideaki Iida, Munazah Andrabi, Jeremy M. Boss, Katsushi Yamaguchi, Shuji Shigenobu, Hisato Kondoh

**Affiliations:** 1 Graduate School of Frontier Biosciences, Osaka University, Yamadaoka 1-3, Suita, Osaka 565-0871, Japan; 2 Department of Developmental Biology, Graduate School of Medical Sciences, Kyushu University, 3-1-1 Maidashi, Higashi-ku, Fukuoka 812-8582, Japan; 3 Faculty of Life Sciences, Kyoto Sangyo University, Motoyama, Kamigamo, Kita-ku, Kyoto 603-8555, Japan; 4 Department of Microbiology and Immunology, Emory University, Atlanta, GA 30322, USA; 5 Functional Genomics Facility, National Institute for Basic Biology, Okazaki, Aichi 444-8585, Japan

**Keywords:** ZIC2, POU3F1, OTX2, ESC, EpiSC, Mouse

## Abstract

To obtain insight into the transcription factor (TF)-dependent regulation of epiblast stem cells (EpiSCs), we performed ChIP-seq analysis of the genomic binding regions of five major TFs. Analysis of *in vivo* biotinylated ZIC2, OTX2, SOX2, POU5F1 and POU3F1 binding in EpiSCs identified several new features. (1) Megabase-scale genomic domains rich in ZIC2 peaks and genes alternate with those rich in POU3F1 but sparse in genes, reflecting the clustering of regulatory regions that act at short and long-range, which involve binding of ZIC2 and POU3F1, respectively. (2) The enhancers bound by ZIC2 and OTX2 prominently regulate TF genes in EpiSCs. (3) The binding sites for SOX2 and POU5F1 in mouse embryonic stem cells (ESCs) and EpiSCs are divergent, reflecting the shift in the major acting TFs from SOX2/POU5F1 in ESCs to OTX2/ZIC2 in EpiSCs. (4) This shift in the major acting TFs appears to be primed by binding of ZIC2 in ESCs at relevant genomic positions that later function as enhancers following the disengagement of SOX2/POU5F1 from major regulatory functions and subsequent binding by OTX2. These new insights into EpiSC gene regulatory networks gained from this study are highly relevant to early stage embryogenesis.

## Abstract (precis)

**Summary:** Surveying the binding profiles of the key transcription factors ZIC2, OTX2, SOX2, POU5F1 and POU3F1 in mouse epiblast stem cells reveals distinct ZIC2-OTX2-centered gene regulation from that in embryonic stem cells.

## INTRODUCTION

Mammalian somatic cells originate from the epiblast in postimplantation embryos. The success in establishing epiblast stem cells (EpiSCs) from the epiblasts of egg-cylinder-stage mouse embryos (Brons et al., 2007; Tesar et al., 2007; Sumi et al., 2013) created new opportunities for investigating the gene regulatory networks in the epiblast and for deriving specific somatic cell lineages (Iwafuchi-Doi et al., 2011, 2012; Acampora et al., 2013; Factor et al., 2014; Kojima et al., 2014; Matsuda and Kondoh, 2014; Tsakiridis et al., 2014; Henrique et al., 2015; Li et al., 2015; Song et al., 2016). EpiSCs can be maintained in feeder-free culture conditions supplemented with activin, which is a Nodal replacement, and bFGF (FGF2) (Brons et al., 2007; Iwafuchi-Doi et al., 2012). They can also be directed towards development into a variety of specific somatic lineages by removing activin and/or manipulating other cell signaling systems (Brons et al., 2007; Tesar et al., 2007; Iwafuchi-Doi et al., 2012; Kojima et al., 2014; Matsuda and Kondoh, 2014; Tsakiridis et al., 2014; Li et al., 2015). For example, simple removal of activin/Nodal signaling directs EpiSCs to develop into anterior neural plate (ANP) cells (Iwafuchi-Doi et al., 2012).

A promising approach to investigate gene regulatory networks is to determine the genomic binding sites of various transcription factors (TFs) using ChIP-seq. In a previous study, we identified ZIC2, OTX2, SOX2, POU5F1 and POU3F1 as major regulators in EpiSCs and during the transition to ANP. These TFs change their anteroposterior localization in the mouse epiblast after embryonic day (E) 6.5, and their overexpression and/or shRNA-mediated knockdown strongly impacts the downstream TFs involved in the derivation of various somatic lineages (Iwafuchi-Doi et al., 2012). OTX2 has also been emphasized as a regulator of early stage embryogenesis and embryonic stem cells (ESCs) (Acampora et al., 2013; Buecker et al., 2014; Yang et al., 2014). An additional focus in this present analysis is to investigate whether SOX2 and POU5F1 share the same genomic targets in mouse and human ESCs, which also express these TFs. Here, we used ChIP-seq technology to determine the genomic binding regions for these TFs to gain a deeper insight into their roles in early stage embryogenesis.

To perform parallel ChIP-seq analysis on a number of TFs with comparable efficiency, we developed a simplified version of the ChIP-seq technique that uses biotinylated TFs (de Boer et al., 2003; Kim et al., 2009). This circumvents the problem caused by antibody-dependent variations in ChIP-seq efficiency and decreases the technical hurdles inherent in conventional ChIP-seq procedures that include cell fractionation. In brief, single expression vectors to achieve *in vivo* biotinylation of a TF were transfected into EpiSCs at high efficiency, albeit under conditions that avoid overdosing cells with exogenous TFs and that maintain the epiblast state. Cell chromatin was then fixed with formaldehyde and whole-cell lysates, rather than isolated nuclei, were processed for the isolation of biotinylated TF-DNA complexes.

The parallel ChIP-seq analysis for five TFs revealed the following new features about the TF binding events. (1) Throughout the entire genome, genomic domains rich in ZIC2 binding of megabase (Mb) scale and those rich in POU3F1 binding alternate, with strong correlation with gene distribution. This presumably reflects the loose clustering of two types of regulatory sequences that act at a short distance and at a long distance from the gene, reflecting the characters of bound TFs. (2) Mouse ESCs and EpiSCs share their SOX2 and POU5F1 binding sites in limited proportions, reflecting the shift in the TFs of major regulatory functions from SOX2/POU5F1 in ESCs to ZIC2/OTX2 in EpiSCs. This shift in the major acting TFs appears to be primed by binding of ZIC2 at relevant genomic positions in ESCs. Thus, the ChIP-seq data on the five TFs in EpiSCs provide new genomic resources that can be utilized in studies on the regulation of stem cells of early stage embryogenesis.

## RESULTS AND DISCUSSION

### Fundamentals of the ChIP-seq procedure using *in vivo* biotinylated TFs

#### Expression of *in vivo* biotinylated TFs

ChIP-seq using a biotinylated TF requires the expression of an exogenous TF fused to a biotin ligase recognition peptide (BLRP) that is biotinylated *in vivo* by co-expressed *E. coli*-derived biotin ligase (BirA) (de Boer et al., 2003). Previously reported ChIP-seq methods using biotinylated TFs employed complex procedures that included the identification of stable transfectant clones with an appropriate level of exogenous TF expression (Kim et al., 2009). We chose transient transfection of an EpiSC population to ascertain that ChIP-seq analyses for a series of TFs were all performed on equivalent cell populations, avoiding the risk of isolating cell variants during cloning. This procedure took advantage of the high efficiency (75-95%; Fig. S1A) (Iwafuchi-Doi et al., 2012) of the transfection protocol that we developed.

**Fig. 1.**
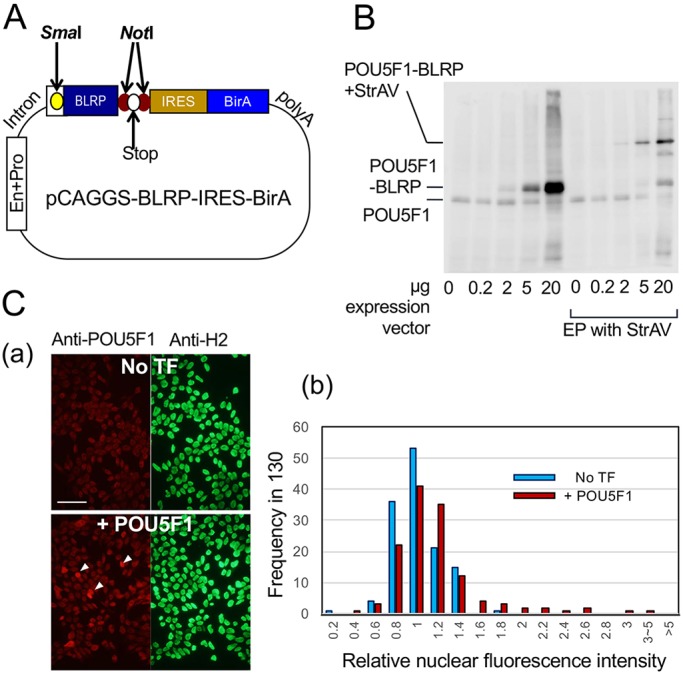
**Expression of biotinylated POU5F1 in transfected EpiSCs.** (A) Schematic of the pCAGGS-BLRP-IRES-BirA vector (not to scale). En, enhancer; Pro, promoter; *Sma*I, the insertion site for the ORF encoding the C-terminal fusion of BLRP; *Not*I, a pair of sites encompassing a stop codon that form the insertion site for the ORF encoding the N-terminal fusion of BLRP. (B) Immunoblots using an anti-POU5F1 antibody comparing the expression of exogenous (BLRP-tagged) and endogenous POU5F1 with increasing amounts of transfected expression vector. The right half of the panel shows the results of electrophoresis (EP) with addition of streptavidin (StrAV), which causes a supershift of biotinylated POU5F1-BLRP. (C) (a) POU5F1 (left) and histone H2 (right) immunofluorescence in the EpiSC population transfected with POU5F1-BLRP compared with the untransfected control. Arrowheads indicate cells expressing high levels of nuclear POU5F1. Scale bar: 50 µm. (b) Variation of POU5F1 immunofluorescence intensities over 130 nuclei in representative fields as assessed using ImageJ and normalized using histone H2 fluorescence. The average immunofluorescence in untransfected nuclei was set as 1.

By transfection of the pCAGGS-BLRP-IRES-BirA vector (Fig. 1A), TFs with BLRP added at their C-terminus were synthesized in EpiSCs, which were ∼2 kDa larger than endogenous TFs, as exemplified by POU5F1-BLRP detected in the western blot (Fig. 1B). Biotinylation of BLRP-tagged POU5F1 was confirmed by supershifting of the electrophoresed protein position following the addition of streptavidin to the SDS-PAGE samples (Viens et al., 2004) (Fig. 1B). In a transactivation assay using the nestin (*Nes*) enhancer, the BLRP-biotin-tagged SOX2 and POU5F1 showed full activity (Fig. S1B). As shown in our previous study, the expression of all five TFs in EpiSCs 24 h after the transfection of shRNA vectors was strongly downregulated (Iwafuchi-Doi et al., 2012), indicating rapid turnover of these TFs, which allows the access of exogenous TFs to their binding sites.

#### Exogenous TF expression at a physiological level

As shown in Fig. 1B, the exogenous TF expression level was controlled by the DNA input level; an input of 5 µg vector DNA per 10-cm diameter culture dish was chosen as standard for transfection to achieve a physiological level of exogenous TF expression.

TF expression levels in individual cell nuclei of POU5F1-BLRP-transfected cells were assessed by POU5F1 immunofluorescence intensity (Fig. 1Ca), which were normalized for histone H2 immunofluorescence of the same nuclei. The results are presented relative to the average fluorescence intensity of untransfected EpiSCs, which was set to 1 (Fig. 1Cb). A shift in nuclear POU5F1 fluorescence intensity to a higher level was noted in the transfected population. In addition, some of the transfected cells expressed higher levels of POU5F1 than observed in untransfected cells (Fig. 1Ca, arrowheads). However, a large majority (95%) of transfected cells had less than twice the average amounts of nuclear POU5F1, with an average increase of 119% in the transfected cells relative to untransfected cells. A higher level of exogenous TF apparent in the immunoblot data using whole cells (Fig. 1B) may be explained by a contribution from cytoplasmic POU5F1.

Analogous data for other TFs generated in parallel experiments are shown in Fig. S1C. Because the same amounts of biotinylated TF expression vector DNA were used for all transfections, the extent of the shift to higher expression levels would be expected to depend on the endogenous TF expression levels. ZIC2 appeared to be expressed at high levels endogenously, and the impact of exogenous ZIC2 expression was minimal, with only a 10% increase, on average, in the immunofluorescence of nuclear ZIC2 (plus ZIC3, which is also recognized by the anti-ZIC2 antibodies). In OTX2-transfected and SOX2-transfected cells, exogenous TF expression increased the nuclear TF levels only to 137% and 130%, respectively, relative to untransfected cell populations. By contrast, POU3F1 appeared to have a relatively low endogenous expression level. Even in this case, the average POU3F1 levels in transfected cell nuclei were estimated to be 252% of normal, and the majority (68%) of nuclei remained with the POU3F1 level within the range of 200% of untransfected cell average.

To confirm that exogenous TF expression does not interfere with the EpiSC state, we analyzed NANOG expression levels in the transfected populations. Even in untransfected EpiSCs, NANOG expression showed variation in its levels, which were divided into brighter and dimmer nuclear populations using a threshold (Fig. S1Da). These populations did not change significantly after exogenous expression of the aforementioned TFs (Fig. S1Db). Analogous analysis of POU5F1 expression in the TF-transfected populations (other than POU5F1 transfection) did not show transfection-dependent changes (data not shown). These analyses indicated that transfection with any of the TFs used for the ChIP-seq analysis did not interfere with the EpiSC state.

#### High performance of the ChIP-seq procedure using biotinylated TFs

**Fig. 2.**
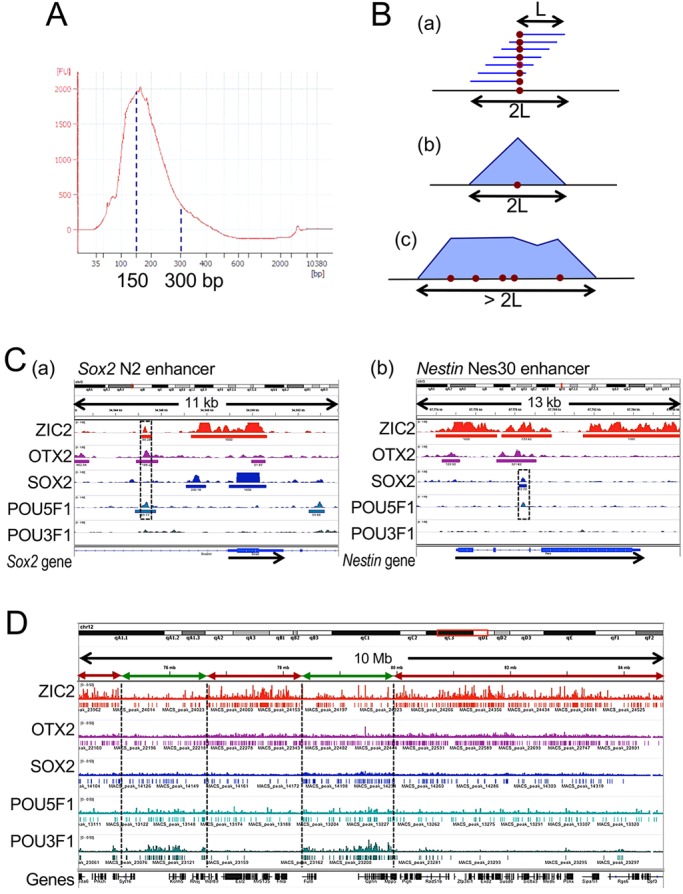
**Relationships of ChIP-seq peak widths with DNA fragment length in ChIP-seq analysis and reiteration of TF binding sites in a short genomic span.** (A) A representative DNA length profile of sheared (Covaris S2-treated) chromatin samples transfected with POU5F1-BLRP, showing a peak at ∼150 bp. (B) Interpretation of ChIP-seq peak widths. If DNA fragments to be used for ChIP-seq analysis have a length of L (a), then ChIP-seq peaks representing single TF bindings will have a peak width of 2L (b). However, reiteration of TF binding in a short genomic span results in wider ChIP-seq peaks, and the occurrence of clustered TF binding sites over a genomic span will cause the appearance of peak ranges (c). (C) Examples of ChIP-seq peaks indicating singlet binding of TFs to the known enhancers *Sox2* N2 (a) and nestin Nes30 (b), as shown using IGV outputs for genomic regions chr12:34,542,000-34,553,800 and chr12:87,773,000-87,786,300, respectively. The direction of gene transcription is indicated by arrows. (a) The N2 enhancer is 5′ of the *Sox2* gene (Iwafuchi-Doi et al., 2012), with ChIP-seq peaks formed at the same position (boxed), indicating the co-binding of ZIC2, OTX2 and POU5F1. The strong *Sox2* peak apparent on the *Sox2* gene itself was due to carryover of the Sox2-BLRP vector. (b) SOX2 and POU5F1 ChIP-seq peaks formed on the Nes30 enhancer (Tanaka et al., 2004) (boxed). (D) IGV outputs of a representative 100 Mb genomic region from chromosome 12 (chr12:74,400,000-84,700,000), showing the TF-dependent arrangements of ChIP-seq peaks for ZIC2, OTX2, SOX2, POU5F1 and POU3F1. MACS annotated peaks are indicated below the Bowtie mapped reads. The approximate boundaries between the regions dominated by clustered ZIC2 (red arrows) and POU3F1 (green arrows) peaks are shown.

One day after transfection with a biotinylated TF-BLRP expression vector for 8 h, the culture was processed for chromatin fragmentation using whole lysate of formalin-fixed cells, yielding chromatin fragments with an average DNA length of 150 bp (Fig. 2A). The TF-BLRP-bound chromatin fragments were extracted using streptavidin-linked magnetic beads under stringent wash conditions (Table S1). This step was followed by standard ChIP-seq procedures. Peak calling using MACS1.4 identified 60,000-120,000 ChIP-seq peaks (Table S2).

### Characterization of ChIP-seq peaks

#### ChIP-seq peak widths

As explained in Fig. 2B, ChIP-seq using a DNA fragment size L will yield a peak width of 2L for singlet binding sites. Considering the modal DNA fragment size of 150 bp and 92nd percentile fragment length of 300 bp used in our ChIP-seq analysis (Fig. 2A), peak widths of less than 500 bp will indicate the occurrence of singlet binding sites. The larger peak widths thus indicate the occurrence of multiple TF binding sites at relatively short genomic intervals that do not separate into individual peaks.

#### ChIP-seq peaks on known enhancers

We evaluated the ChIP-seq peaks obtained using our new procedure by analyzing known TF binding sites: the *Sox2* upstream N2 enhancer, which is activated via co-binding by ZIC2, OTX2 and a POU factor (Iwafuchi-Doi et al., 2011); and the Nes30 enhancer in the second intron of *Nes*, which is known to bind SOX2 and a POU factor in ESCs and neural stem cells (Tanaka et al., 2004; Lodato et al., 2013). These enhancers were indeed bound by the expected TFs, forming overlapping singlet ChIP-seq peaks (Fig. 2C).

#### Enriched DNA sequence motifs in ChIP-seq peaks

The sequences of ChIP-seq peaks of 400-1000 bp with a cumulative length of 30-50 Mb were analyzed using MEME-ChIP (Machanick and Bailey, 2011) to evaluate the enrichment of sequence motifs. The results, as summarized in Fig. S2A, indicate that the ChIP-seq peaks were enriched with sequence motifs relevant for TF binding: ZIC2 binding sequences characterized by interruption of G/C stretches by A/T bases (Lim et al., 2010; Jolma et al., 2013); OTX2 binding sequence approximated by GGTAAT (Briata et al., 1999; Jolma et al., 2013); SOX2 binding motifs represented by ACAA[A/T][A/G] (Kamachi and Kondoh, 2013; Kondoh and Kamachi, 2015); and POU class TF binding motifs of ʻoctamer' consensus sequence ATGCA[A/T]AT (Jolma et al., 2013) with allowance of two base deviations (Kamachi and Kondoh, 2013).

### Alternating pattern of Mb-scale genomic domains rich in ZIC2 and those rich in POU3F1 peaks

We inspected the genomic distribution of the annotated ChIP-seq peaks over the mouse genome using Integrative Genome Viewer (IGV) (Robinson et al., 2011) with a scrolling unit of several Mb. This visual inspection indicated the following features (see Fig. 2D, for example). Genomic domains of a Mb scale that are rich in ZIC2 peaks and those rich in POU3F1 peaks alternate with one another. The distribution of ChIP-seq peaks of three other TFs was more uniform, although OTX2 peaks tended to be associated with ZIC2 peaks, and SOX2 and POU5F1 peaks with POU3F1 peaks. In addition, ZIC2 peak-rich domains were also rich in genes.

**Fig. 3.**
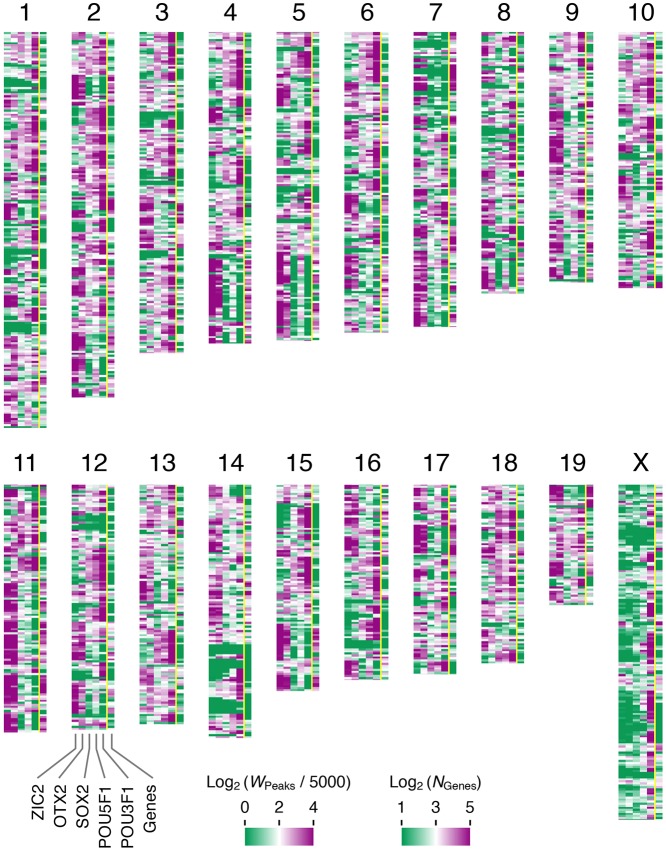
**Heat map representation of ChIP-seq peak and gene densities in 1 Mb genomic intervals.** The mouse mm9 genomic sequence of all chromosomes was divided into segments of 1 Mb and then the segments without any ChIP-seq peaks were removed. The sum of the peak widths for each TF per Mb segment (*W*_Peaks_) divided by 5000, or the number of RefSeq gene (Pruitt et al., 2014) TSSs per segment (*N*_Genes_), were ranked on a logarithmic scale using the heat map over individual chromosomes (shown oriented with centromere sides to the top).

To substantiate these observations, we divided the genomic sequence of all chromosomes into 2582 segments (chromosomal segments of 1 Mb) and scored the sum of the peak widths for each TF per Mb (peak densities) or the number of genes in a segment. The distribution of the ChIP-seq peak densities for individual TFs over the genome is summarized as a heat map in Fig. 3 and is compared with the distribution of the genes. The heat map data were consistent with the conclusions derived on the basis of the IGV-based visual inspection of the ChIP-seq peak distribution (Fig. 2D).

To further confirm our observations, we performed the following statistical analysis. After normalizing the average peak density to a value of 1 for all TFs, the ranges of variation in peak density in individual Mb segments were plotted for the five TFs (Fig. S2B). The peak density per Mb genomic segment for ZIC2 and POU3F1 showed a very broad distribution from very high (>5) to very low (<0.1) values, corresponding to peak-rich and peak-sparse genomic domains. By contrast, the ChIP-seq peak densities for OTX2, SOX2 and POU5F1 had a much narrower distribution.

#### High clustering tendency of ZIC2 and POU3F1 binding sites

ChIP-seq peaks of ZIC2 and POU3F1 were often clustered more densely than those of the other three TFs. This was confirmed by the peak gap size analysis shown in Fig. S2C, gap size being the distance between the midpoints of two adjacent peaks. The analysis indicated that for ZIC2 and POU3F1 the peak gap size was distributed more broadly, with the modal gap size one order of magnitude shorter than predicted from random occurrence of the peaks. However, this feature was not apparent for OTX2, SOX2 or POU5F1 peaks.

An additional effect of dense clustering of TF binding sites is that individual ChIP-seq peaks corresponding to the TF binding sites fail to separate and instead form a peak range that covers a long genomic span. The analysis of ChIP-seq peak widths for the TFs (Fig. S2D) indicated that ZIC2 and POU3F1 binding sites reiterate frequently, resulting in >2000 bp peak ranges occupying 20% and 29% of peaks, respectively. By contrast, 60% and 70% of OTX2 and SOX2 binding sites, respectively, were in ChIP-seq peaks <1000 bp, indicating that they very frequently occur as singlet binding sites. The distribution of POU5F1 binding sites appears to be intermediate between these two cases.

#### TF-dependent proximity of ChIP-seq peaks to transcription start sites (TSSs)

The distance of ChIP-seq peaks from neighboring genes classified the TFs into two classes (Fig. S3A). The majority (55%) of ZIC2 and OTX2 peaks were within 50 kb of a TSS. By contrast, more than 80% of the SOX2, POU5F1 and POU3F1 peaks were at least 50 kb away from a TSS. These observations indicated that ZIC2 and OTX2 most often function by binding to gene-proximal regulatory sequences, whereas SOX2 and POU factors primarily function via gene-remote regulatory sequences.

The ChIP-seq peaks within 5 kb of a TSS were further characterized using aggregation plots (Fig. S3B). The local TSS density per ChIP-seq peak at 5 kb points averaged over the genome varied depending on the TF. Approaching the ChIP-seq peak position, strong positive TSS peaks (maximum ∼0.01) occurred with ZIC2. By contrast, POU3F1 and POU5F1 peaks, starting from already low TSS densities, further decreased at the peak position, indicating that the overlap of a POU3F1 or POU5F1 peak with a TSS is very rare. The aggregation plots using OTX2 and SOX2 had bumps at ∼1000 bp from the ChIP-seq peaks, but depressions at these ChIP-seq peak midpoints, indicating that OTX2 or SOX2 ChIP-seq peaks are more enriched at ∼1000 bp from TSSs.

The differences in the positioning of TF binding sites, either in close proximity to or distant from genes (Fig. S4), and their tendency for clustering (Fig. S2B,C) appear to form the basis of the alternating patterns of genomic domains rich in ZIC2 and those rich in POU3F1 peaks (Fig. 3). Whether this type of patterning is unique to EpiSCs and whether the patterns vary with stem cell type warrant future studies.

### ChIP-seq peak overlap in EpiSCs indicates two TF groups

#### Two groups of TFs that show a high frequency of in-group ChIP-seq peak overlap

**Fig. 4.**
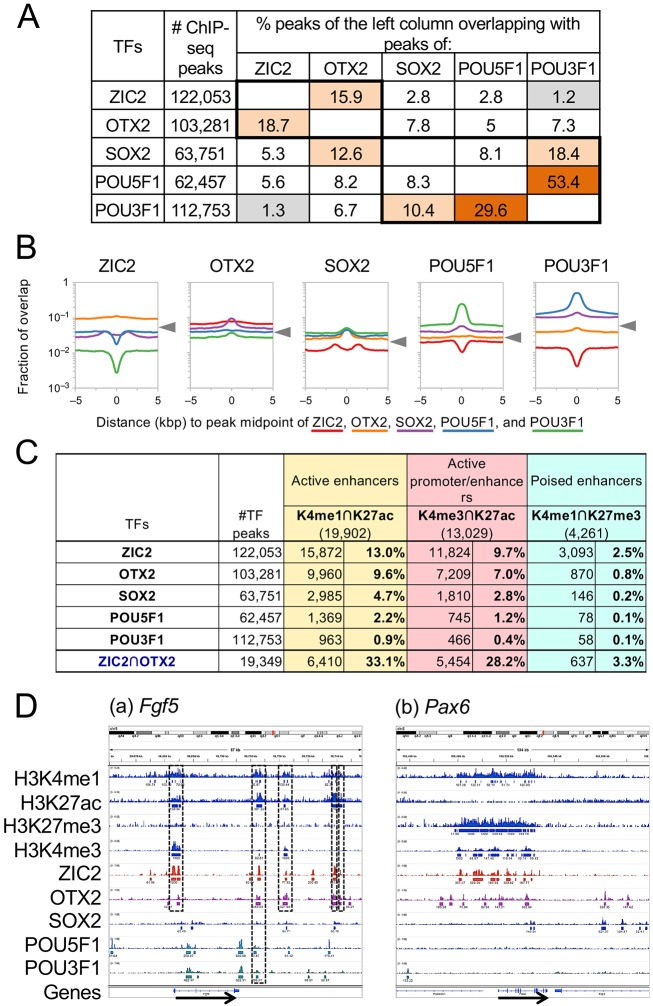
**Statistics on the overlap of ChIP-seq peaks.** (A) The frequency of overlap of MACS annotated peaks. Overlap rates higher than 10% and lower than 2% are highlighted in orange and gray, respectively. High-frequency peak overlaps in ZIC2-OTX2 and SOX2-POU TF groups are indicated by thick-lined boxes. (B) Aggregation plots illustrating the fraction of relative midpoint positions of the ChIP-seq peaks of target TFs (as indicated by color codes) that overlap with the peaks of the anchor TF shown at the top. Ordinate shows the fraction of overlap on a logarithmic scale measured in 100 bp bins. The levels of the plot assuming random distribution of the TF peaks are indicated by arrowheads. (C) The fraction of TF ChIP-seq peaks overlapping with genomic regions harboring specific histone H3 modification signatures. (D) Comparison of ChIP-seq peaks of TF binding and the locations of histone H3 modifications around the genes representative of specific cell lineages. IGV outputs of the genomic regions chr5:98,661,300-98,748,500 (a) and chr2:105,475,120-105,578,450 (b) are shown. The genes and their direction of transcription are indicated by arrows. (a) *Fgf5*, expressed in EpiSCs. Overlap of TF peaks and modified histone H3 peaks is emphasized by boxes. (b) *Pax6*, representing anterior neural plate development.

The majority of TFs function via interaction with other TFs. We investigated the overlap of ChIP-seq peaks between two TFs, as summarized in Fig. 4A. The following features of overlapping ChIP-seq peaks were noted. (1) The overlap between ZIC2 and POU3F1 peaks was minimal (1.2-1.3%). (2) A significant proportion (15.9-18.7%) of ZIC2 and OTX2 peaks overlapped, suggesting their functional interactions. (3) POU5F1 and POU3F1 peaks overlapped at high frequency (30-53%), as expected from their sharing of similar *in vitro* binding sequences (Jolma et al., 2013). However, the remaining ChIP-seq peaks were unique to each POU factor. (4) SOX2 ChIP-seq peaks overlapped most frequently with POU3F1 peaks (17.5-10.4%) and less so with POU5F1 peaks (7.7-8.3%). These rates of overlap were much lower than in ESCs, where the majority of SOX2 and POU5F1 peaks overlap (Chen et al., 2008) (see also Fig. 6A). (5) ChIP-seq peak overlap was limited between the TF groups ZIC2 plus OTX2 versus SOX2 plus POUs, except for 12% of SOX2 peaks that overlapped with OTX2 peaks.

As the expression levels of TFs among the EpiSC population are not perfectly homogeneous (Fig. 1C, Fig. S1C), we investigated the possibility that EpiSCs comprise alternative subpopulations of high ZIC2 expressers and high POU3F1 expressers, or of POU5F1 expressers and POU3F1 expressers, which might account for the poorly overlapping binding events between ZIC2 and POU3F1, as well as the occurrence of individually unique binding sites for POU5F1 and POU3F1 (Fig. 4A). As shown in Fig. S4, the expression levels of the two sets of TFs were positively correlated (correlation coefficient, *r=*∼0.6), indicating that the ChIP-seq peak overlap patterns shown in Fig. 4A occur in the same cell populations.

We also analyzed the relative positioning of ChIP-seq peak midpoints between a pair of TFs using aggregation plots that are independent of peak widths (Fig. 4B). The target TF-dependent variations of the plots indicated TF pair-dependent association or dissociation of the ChIP-seq peaks, whereas the random occurrence of the peaks would result in convergence of the plots to levels unique to the anchor TFs (indicated by the arrowheads in Fig. 4B). The occurrence of a deep depression at position zero indicated mutually exclusive binding sites between ZIC2 and POU3F1, and to a lesser extent between ZIC2 and POU5F1. Proximal co-occurrence of the binding sites among POU3F1, POU5F1 and SOX2 was also indicated. All aggregation plot data in Fig. 4B supported the conclusions drawn from the ChIP-seq peak overlap data (Fig. 4A).

#### Correlations between TF binding regions and histone modification signatures

We investigated whether the ChIP-seq peaks of the above-mentioned TFs represent regulatory sequences, taking advantage of published ChIP-seq data on histone H3 modifications in EpiSCs (Factor et al., 2014). Generally, active enhancers are marked by a combination of H3K4me1- and H3K27ac-enriched domains, whereas poised enhancers are marked by H3K4me1- and H3K27me3-enriched domains. The H3K4me3/K27ac combination marks active promoters and promoter-proximal enhancers (Ong and Corces, 2012; Calo and Wysocka, 2013).

To evaluate the correlations between TF binding and the combined histone H3 modification signatures, we analyzed the frequency of ChIP-seq peak overlaps between the TF peaks and modified H3 peaks (Factor et al., 2014) (Fig. 4C). Among TFs, ZIC2 binding was the most frequently associated not only with the H3K4me1 plus H3K27ac (active enhancer type) modifications, but also with the H3K4me1 plus H3K27me3 (poised enhancer type) modifications, indicating that ZIC2 binding is involved in both activating and repressing gene transcription. Importantly, as much as 33% of the ZIC2-OTX2 co-bound peaks overlapped with the H3K4me1 plus H3K27ac peaks, suggesting that the combination of ZIC2 and OTX2 mainly acts as a transcriptional activator in EpiSC gene regulatory networks. Aggregation plots showing the distance relationships of histone modification signatures relative to ChIP-seq peak positions also supported the these conclusions (Fig. S3C).

Local TF binding and histone H3 modification profiles also supported the above proposition, as exemplified by the *Fgf5* and *Pax6* loci shown in Fig. 4D. *Fgf5*, a characteristic EpiSC-expressed gene, is activated by ZIC2, OTX2 and POU3F1 (Iwafuchi-Doi et al., 2012), and five overlapping ZIC2 and OTX2 ChIP-seq peaks, one of which was also bound by POU3F1, found near the *Fgf5* gene (Fig. 4Da) were marked by H3K4me1/K27ac modifications, suggesting that these TF binding sites represent *Fgf5* enhancers in EpiSCs. *Pax6* is silent in EpiSCs, but is rapidly activated when ANP development is initiated (Iwafuchi-Doi et al., 2012). The *Pax6* locus in EpiSCs showed several prominent ZIC2 and OTX2 ChIP-seq peaks upstream and in the gene body (Fig. 4Db), which are covered by dense H3K4me1 plus H3K27me3 signatures, indicating the poised state of the enhancers.

**Fig. 5.**
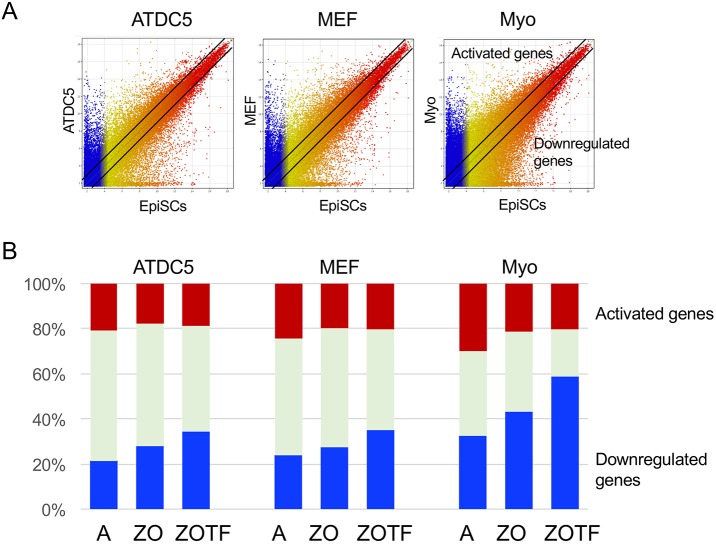
**Changes in gene expression profiles of somatic cells as compared with EpiSCs for genes proximal to ZIC2-OTX2 co-binding regions marked by histone modification signatures as active enhancers.** Microarray data for the chondrogenic cell line ATDC5, MEF (mouse embryonic fibroblasts) and Myo (mouse embryonic primary myoblasts) were compared with those of EpiSCs (Iwafuchi-Doi et al., 2012). The dots are colored according to the levels in EpiSCs. (A) Scatter plots showing the differences in gene expression profiles between EpiSCs and the three somatic cell types. Greater than 2-fold differences compared with EpiSCs were scored as activation or downregulation, as indicated by the lines in the plots. (B) The fraction of activated and downregulated genes for all genes (column A), genes proximal to ZIC2-OTX2 co-bound regions marked by H3K4me1/K27Ac (ZO), and TF genes proximal to ZIC2-OTX2 co-bound regions marked by H3K4me1/K27Ac (ZOTF).

To confirm the involvement of the ZIC2-OTX2-bound genomic regions marked by H3K4me1/K27ac in the activation of proximal genes in EpiSCs, we compared the global gene regulation and the regulation of genes proximal to histone signature-bearing ZIC2-OTX2 peaks in EpiSCs using published microarray expression data of various somatic cells. The underlying rationale is that a significant fraction of ZIC2-OTX2-dependent enhancers in EpiSCs will be discharged from their regulatory function in somatic cells, and hence genes proximal to such enhancers will be more frequently downregulated than unselected genes. We compared the gene expression profile of the chondrogenic cell line ATDC5 (GEO accession GSM1486495) (Sugita et al., 2015), mouse embryo fibroblasts (GSM1079106) (Richter et al., 2013) and mouse embryo primary myoblasts (GSM1541934) with that of EpiSCs (GSM934422/GSM934427) (Iwafuchi-Doi et al., 2012). As shown in Fig. 5, equivalent numbers of genes were either activated or downregulated compared with those in EpiSCs using 2-fold differences as a cut-off for all cell types, among which myoblasts showed the greatest divergence from EpiSCs (33% of genes downregulated and 30% of genes activated). However, when 4722 genes with a TSS within 5 kb of histone signature-bearing ZIC2-OTX2 ChIP-seq peaks were selected, a larger fraction of genes was downregulated (43% for myoblasts) and a smaller fraction of genes was activated (21% for myoblasts) (Fig. 5B, ZO). Moreover, when 291 TF genes within 5 kb of the histone signature-bearing ZIC2-OTX2 ChIP-seq peaks were selected from all TF genes expressed in EpiSCs (Iwafuchi-Doi et al., 2012), a significantly larger fraction of genes was downregulated in the somatic cells (59% for myoblasts) (Fig. 5B, ZOTF). The downregulated TF genes in the ZOTF column included those involved in the regulation of pluripotent stem cells, e.g. *Mycn*, *Nanog*, *Otx2*, *Pou5f1*, *Sall4* and *Sox2*. This analysis confirmed that the ZIC2-OTX2 pair is involved in the activation of genes in EpiSCs, particularly the TF genes.

### Variations in the binding regions of SOX2 and POU5F1 among stem cells in relation to the ZIC2-OTX2 interaction

**Fig. 6.**
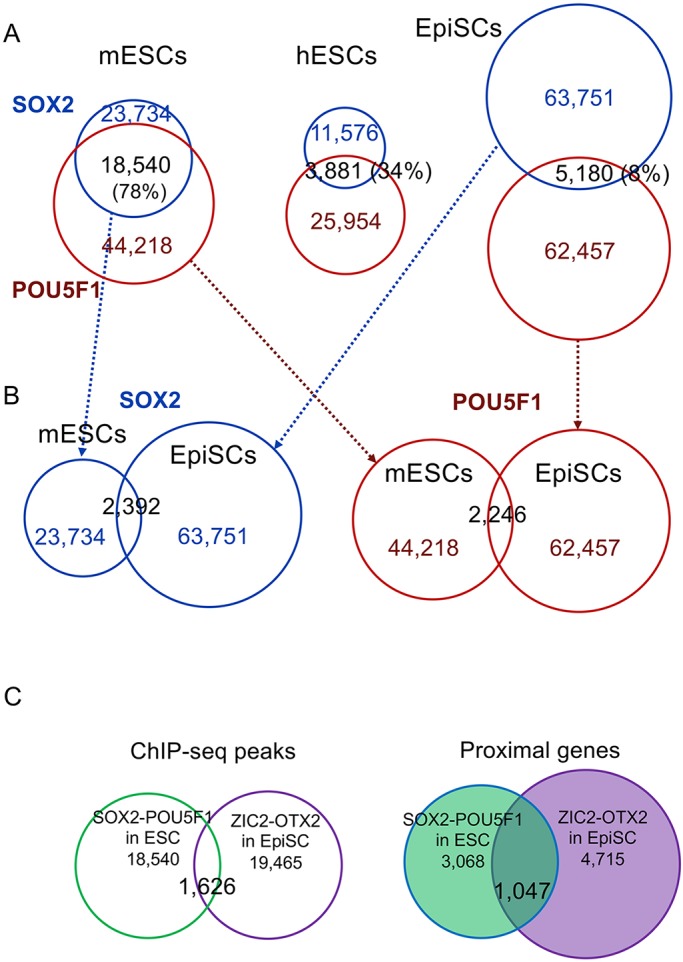
**Variation in SOX2 and POU5F1 ChIP-seq peak overlaps between ESCs and EpiSCs in relation to ZIC2/OTX2 ChIP-seq peaks in EpiSCs.** (A) Venn diagram comparison of SOX2 and POU5F1 ChIP-seq peak overlaps among mouse ESCs, human ESCs and mouse EpiSCs. (B) Overlaps of SOX2 peaks and POU5F1 peaks in mouse ESCs and EpiSCs. (C) Overlap of ChIP-seq peaks for SOX2/POU5F1 in ESCs and for ZIC2/OTX2 EpiSCs (left), and that of genes proximal (<50 kb for the former and <5 kb for the latter) to respective ChIP-seq peaks (right).

It has been shown that the SOX2-POU5F1 TF pair plays major regulatory roles in ESCs (Chen et al., 2008). Although EpiSCs also express high levels of SOX2 and POU5F1 (Brons et al., 2007; Tesar et al., 2007; Iwafuchi-Doi et al., 2012), their genomic binding sites were highly divergent in EpiSCs (Fig. 6A,B). The SOX2 ChIP-seq peaks in ESCs (Lodato et al., 2013) overlapped extensively (78%) with POU5F1 peaks (Fig. 6A). In sharp contrast, only ∼8% of SOX2 and POU5F1 peaks overlapped in EpiSCs (Fig. 4A, Fig. 6A). However, this low rate of overlap was not unexpected because a previous report indicated that the POU5F1 binding genomic sites in human ESCs are highly diversified from mouse ESCs (Fig. 6A) (Kunarso et al., 2010).

A comparison of the SOX2 and POU5F1 ChIP-seq peaks between ESCs and EpiSCs indicated that only 5-10% of the peaks overlapped (Fig. 6B). This analysis was extended to two additional stem cells, namely human ESCs derived from pre-implantation blastocysts but maintained under the culture conditions of EpiSCs, and mouse epiblast-like cells (EpiLCs) that are produced as a transient state when ESCs are directly placed in EpiSC culture conditions, exhibiting some EpiSC characteristics in cell morphology and gene expression, but which only survive for a short time (Hayashi et al., 2011).

Two sets of SOX2 and POU5F1 ChIP-seq peak data for human ESCs (Table S4) were transformed into mouse genome coordinates (mm9), and the overlap frequencies of the human ESC ChIP-seq peaks with those of mouse ESCs and EpiSCs were scored (Tables 1 and 2). The overlap of SOX2 ChIP-seq peaks was ∼10% between mouse ESCs and human ESCs, as well as between human ESCs and mouse EpiSCs (Table 1). However, the overlap of the POU5F1 peaks between mouse and human ESCs was higher (∼23%), whereas that between human ESCs and mouse EpiSCs was much lower (∼3%) (Table 2). The POU5F1 ChIP-seq peak data of EpiLCs (Buecker et al., 2014) indicated a closer relationship to mouse ESCs (Table 2), revealing the following developmental order: mouse ESCs, EpiLCs, human ESCs and then EpiSCs.

**Table 1.**
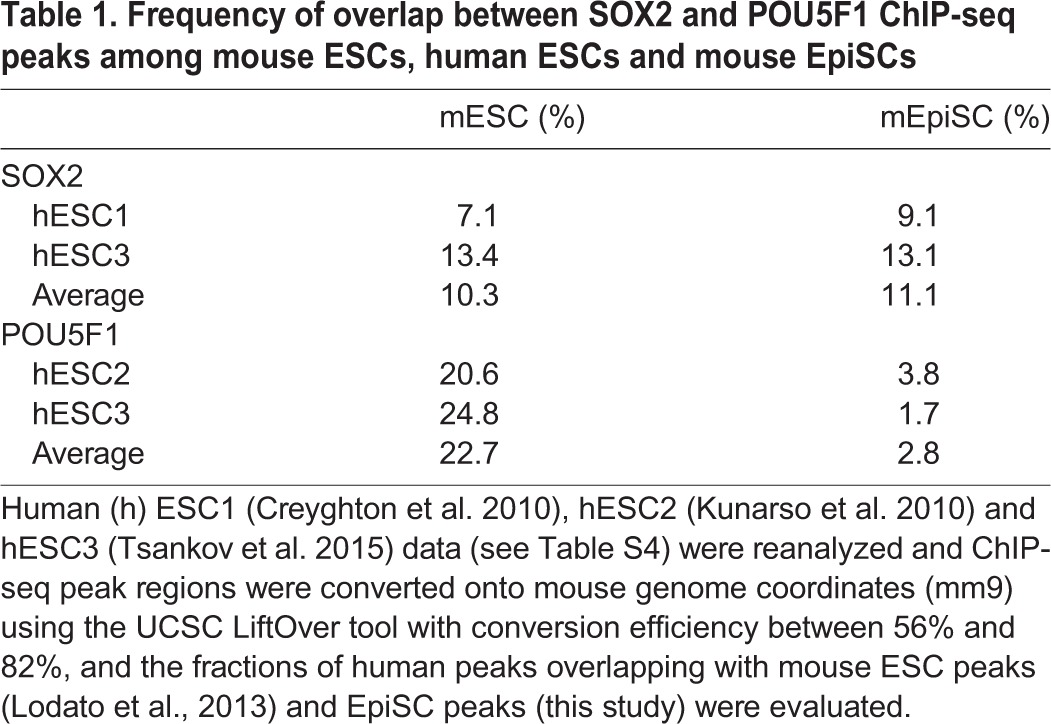
**Frequency of overlap between SOX2 and POU5F1 ChIP-seq peaks among mouse ESCs, human ESCs and mouse EpiSCs**

**Table 2.**
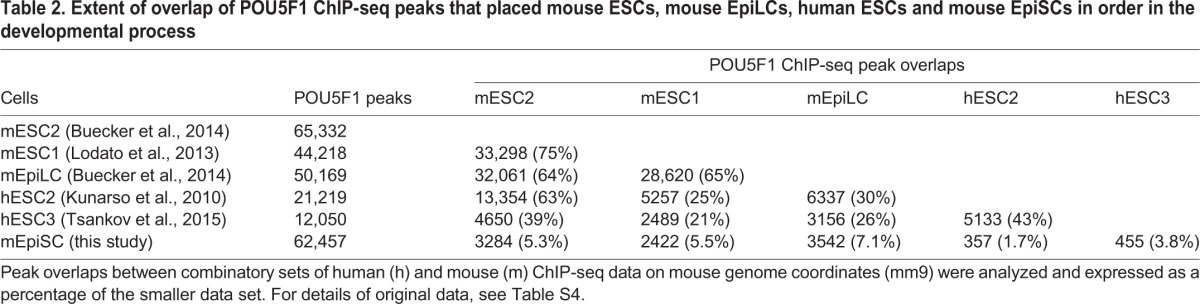
**Extent of overlap of POU5F1 ChIP-seq peaks that placed mouse ESCs, mouse EpiLCs, human ESCs and mouse EpiSCs in order in the developmental process**

Despite the diversity of SOX2 and POU5F1 binding patterns (Fig. 6A,B), the majority of genes expressed in ESCs are nearly equally expressed in EpiSCs (Factor et al., 2014). Factor et al. (2014) also reported that a significant fraction of the genomic positions for enhancer candidates, marked by H3K27ac, differ between these two cell types. These observations suggest that these two enhancer systems, one of which is dependent on SOX2-POU5F1 interaction in ESCs whereas the other is dependent on ZIC2-OTX2 interaction in EpiSCs, give rise to similar gene expression profiles.

Overlap of SOX2/POU5F1-co-bound regions in ESCs (18,540) and ZIC2/OTX2-co-bound regions in EpiSCs (19,465) was limited to less than 10% (1626) (Fig. 6C). This was also true when histone H3K4me1/K27ac-marked active enhancer regions were analyzed: 5977 SOX2/POU5F1-co-bound and H3K4me1/K27ac-marked regions were present in ESCs, and 6410 ZIC2/OTX2-co-bound and H3K4me1/K27ac-marked regions were present in EpiSCs, with an overlap of only 472 regions. Despite this finding, a much higher frequency of overlap (1047 genes, 34%) was observed between genes proximal to the SOX2/POU5F1-co-bound regions with active enhancer signatures in ESCs, and those proximal to the marked ZIC2/OTX2-co-bound regions in EpiSCs (Fig. 6C). The expected overlap of the same numbers of randomly selected RefSeq genes was 398. These data support the above-mentioned model that different enhancers that are dependent on different TFs can activate similar gene sets in ESCs and EpiSCs.

**Fig. 7.**
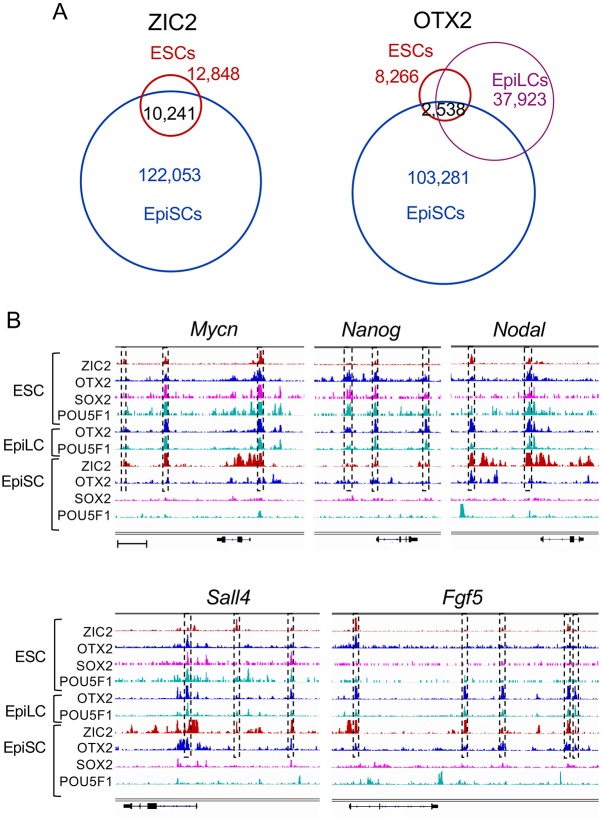
**Cell type-dependent variation in TF binding at specific genomic regions.** (A) Venn diagram comparison of the ChIP-seq peak positions for ZIC2 and OTX2 in ESCs and EpiSCs, showing peak overlaps. For OTX2, data for EpiLCs are included. Data sources are indicated in Table S4. (B) IGV diagrams showing genomic regions of the pluripotency-specific genes *Mycn* (chr12:12,926,000-12,961,000), *Nanog* (chr6:122,647,000-122,667,000), *Nodal* (chr10:60,865,000-60,890,000) and *Sall4* (chr2:168,571,500-168,623,000) and of the EpiSC-specific gene *Fgf5* (chr5:98,658,500-98,747,000). Scale bar indicates 5 kb genomic span for upper panels and 7.5 kb for lower panels. Dashed boxes indicate the genomic regions where ZIC2 binding is indicated by ChIP-seq peaks in ESCs and EpiSCs. These regions are also bound by other TFs in ESCs, EpiLCs or EpiSCs, but rarely by SOX2 or POU5F1 in EpiSCs.

We used published ChIP-seq data to examine ZIC2 and OTX2 binding genomic regions in ESCs and EpiLCs. There were fewer ChIP-seq peaks reported for these TFs in ESCs than in EpiSCs, presumably reflecting increase in their binding sites after exit from ESCs, as previously shown for OTX2 (Yang et al., 2014). Comparing the ZIC2 ChIP-seq peaks in ESCs (Luo et al., 2015) with those in EpiSCs, we found that the majority (80%) of ZIC2-bound regions in ESCs were also bound by ZIC2 in EpiSCs (Fig. 7A). However, OTX2 ChIP-seq peaks in ESCs, EpiLCs, and EpiSCs showed more cell type-dependent variations (Fig. 7A), suggesting that at least a fraction of ZIC2 binding sites are already established in the ESCs. It is thus possible that ZIC2 acts as a ʻpioneer factor' (Zaret and Mango, 2016), contributes to organizing ʻseed enhancers' (Factor et al., 2014) or, more generally, primes enhancer activity. It has been shown that ZIC2 in ESCs interacts with nucleosome remodeling and deacetylase (NuRD) complexes (Luo et al., 2015) that are involved in the regulation of ʻbivalent' enhancers in ESCs. These enhancers are generally inactive in most ESCs but are poised for gene activation upon exit from the ESC state (Hu and Wade, 2012; Harikumar and Meshorer, 2015). Therefore, the ZIC2-NuRD interaction might be one mechanism for seeding enhancers.

Factor et al. (2014) also identified 606 pluripotent cell-specific genes that are expressed in both ESCs and EpiSCs but are turned off in various somatic cells. One-quarter of these genes are included in the above-mentioned gene set that is proximal to both putative active SOX2/POU5F1-bound enhancers in ESCs and putative enhancers bound by ZIC2/OTX2 in EpiSCs. As shown in IGV diagrams of the genomic regions surrounding such genes, as exemplified by *Mycn*, *Nanog*, *Nodal* and *Sall4* (Fig. 7B), ZIC2-OTX2 binding regions in EpiSCs were, in the majority of cases, bound by ZIC2 and also by SOX2 plus POU5F1 in ESCs. Very few of these ZIC2-bound regions are bound by SOX2 or POU5F1 in EpiSCs. Some, but not all, ZIC2 ChIP-seq peak regions were also bound by OTX2 in ESCs. On comparing the ESC ChIP-seq data for OTX2 binding (Buecker et al., 2014), ZIC2 binding, and histone H3 modifications (Luo et al., 2015), 16% of ZIC2 peaks were estimated to overlap with OTX2 peaks, among which 75% were marked by H3K4me1/K27ac modifications for active enhancers. This suggests that, although a large fraction of ZIC2 binding sites in ESCs might be involved in NuRD-mediated bivalent enhancer regulation, most of the ZIC2-OTX2 co-bound enhancers are already active in ESCs. It is thus possible that early ZIC2 binding, to the same or nearby enhancer regions, might maintain the activity of those enhancer regions, or activate ZIC2-bound cryptic enhancers, once SOX2 and POU5F1 are disengaged from their major regulatory functions. In the EpiLCs, the SOX2/POU5F1-bound regions in ESCs were, in most cases, bound by OTX2 and POU5F1, as reported (Buecker et al., 2014). It will be interesting to discover more about ZIC2 and SOX2 binding regions in cells of this intermediate state.

ZIC2 binding-dependent enhancer priming in ESCs for later enhancer activity appears to apply to a wider range of genes than those that are pluripotency specific. Even in the EpiSC-specific *Fgf5* gene, all five putative enhancers were bound by ZIC2 and not by OTX2 in ESCs (Luo et al., 2015), by OTX2 and POU5F1 in EpiLCs (Buecker et al., 2014), and by ZIC2 and OTX2 in EpiSCs (Fig. 7B). Luo et al. (2015) reported that whereas ZIC2 function in ESCs is dispensable, its downregulation in ESCs affects later developmental processes. These findings strongly support an enhancer-priming function of ZIC2 in ESCs in the regulation of genes activated during later developmental stages.

### Conclusions

In this study we developed an efficient procedure for ChIP-seq analysis utilizing *in vivo* biotinylated TFs. We applied this procedure to investigate genomic binding regions for TFs ZIC2, OTX2, SOX2, POU5F1 and POU3F1, which have been shown to be involved in EpiSC maintenance and somatic cell lineage derivation (Iwafuchi-Doi et al., 2012).

ChIP-seq peak distributions of the Mb order revealed alternating genomic domains rich in both ZIC2 peaks and genes, and domains rich in POU3F1 peaks but sparse in genes. This domain organization presumably reflects the clustering of regulatory regions that have short- and long-range effects, which are dependent on the binding of ZIC2 and POU3F1, respectively. It is an intriguing question whether the regulatory region clustering is associated with higher-order subgenomic activities.

ChIP-seq peaks of ZIC2 and OTX2 overlapped most extensively, and their overlapping peaks were strongly associated with histone modification signatures of active or poised enhancers, suggesting functional cooperation of these TFs. SOX2 and POU factors formed a second group of overlapping peaks. The comparison of gene expression patterns between EpiSCs and somatic cells indicated that enhancers bound by ZIC2 and OTX2 prominently regulate TF genes in EpiSCs.

Despite the similarity of the overall gene expression profile and the high expression of SOX2 and POU5F1 in each cell type, the SOX2 and POU5F1 binding regions in EpiSCs were strikingly different from those in mouse ESCs. Although the major acting TFs appeared to shift from SOX2/POU5F1 in ESCs to ZIC2/OTX2 in EpiSCs, similar sets of genes were regulated in both cell types. This shift in the major acting TFs appears to be primed by binding of ZIC2 at relevant genomic positions in ESCs that later function as enhancers.

## MATERIALS AND METHODS

### EpiSC culture and expression of biotinylated TFs

An EpiSC line (Tesar et al., 2007; Iwafuchi-Doi et al., 2012; Factor et al., 2014) was maintained in feeder-free conditions as described previously (Iwafuchi-Doi et al., 2012). The cells were processed for immunofluorescence of TFs using the IgGs listed in Table S3, including non-commercial rabbit anti-ZIC2 (which also recognizes ZIC1 and ZIC3) (Inoue et al., 2007), and duplicate samples. Expression of all hallmark TFs of EpiSCs and nuclear DAPI staining (Fig. 1, Figs S1 and S3) confirmed proper culture conditions. Images were taken using an Axioplan 2 microscope (Zeiss) and DP71 camera (Olympus) and measured for nuclear fluorescence intensities using ImageJ (Schneider et al., 2012). To express BLRP-tagged TFs and *E. coli*-derived biotin ligase (BirA) from the same plasmid, the BLRP-IRES-BirA module of pREP4-BLRP-TEV-His6 (Lai et al., 2011; Majumder et al., 2014) was transferred to the pCAGGS vector (Sawicki et al., 1998) to produce pCAGGS-BLRP-BirA (Fig. 1A), and modified so that the BLRP could be added to either the N-terminus or C-terminus of the TF depending on the ORF insertion site. In this study, BLRP was added at the C-terminus of TFs. The entire nucleotide sequence of pCAGGS-BLRP-BirA is provided in Fig. S5.

### ChIP-seq procedure

A suspension of Accutase-dissociated EpiSCs was plated on a 10-cm diameter dish and immediately transfected, using Lipofectamine 2000 (ThermoFisher), with 5 µg pCAGGS-BLRP-BirA plasmid to express a BLRP-tagged TF. After 8 h, the culture medium was refreshed to remove the transfection reagents and 50 µg/ml biotin was added. After 24 h, the cultures were fixed with 1% formalin for 10 min at room temperature, and processed according to the procedure shown in Table S1. Typically, several nanograms of precipitated DNA were obtained from five 10-cm diameter dish cultures. The DNA fragments were ligated with adapters and amplified by ten cycles of PCR using the TruSeq ChIP Sample Prep Kit (Illumina) to prepare libraries for DNA sequence determination using an Illumina HiSeq2500 and single-end reads of 100 bp.

### Analysis of ChIP-seq data

FASTQ sequences were aligned to the mouse mm9 genome sequence using Bowtie2 (Langmead and Salzberg, 2012) and converted to SAM and then BAM files. Then, ChIP-seq peaks were called using MACS1.4 (Zhang et al., 2008), input DNA without ChIP as reference, and the default settings except for band width, which was 150 bases. External data (Table S4) were analyzed using the default settings. The Bowtie-aligned peaks and MACS-determined peak positions were visualized using IGV (Robinson et al., 2011) and ChIP-Atlas (http://chip-atlas.org). The above data processing was performed using the software package SraTailor (Oki et al., 2014). Peak overlaps were analyzed using Galaxy (Giardine et al., 2005), and the genomic spans given in BED format were converted to FASTA files using the UCSC Table Browser (Karolchik et al., 2004) in order to be analyzed for sequence motif enrichment by MEME-ChIP (Machanick and Bailey, 2011). To compare human peak data with mouse data, human BED file data were transferred onto the mouse genome coordinates using the LiftOver tool of the UCSC genome browser. Association of ChIP-seq peaks with closest genes was analyzed using GREAT software (McLean et al., 2010), and the overlaps of gene sets were analyzed using BioVenn (Hulsen et al., 2008). Microrarray data were analyzed using GeneSpring 14.5 software (Agilent). The ChIP-seq data reported in this paper were deposited at Gene Expression Omnibus database with accession number GSE74636 (see Table S4).
